# Designing and Evaluation of a Plasmid Encoding Immunogenic Epitopes From *Echinococcus granulosus* Eg95-1-6, P29, and GST Against Hydatid Cyst in BALB/c Mice

**DOI:** 10.1155/japr/1655679

**Published:** 2025-02-24

**Authors:** Sasan Khazaei, Abdolhossein Dalimi, Majid Pirestani, Fatemeh Ghafarifar

**Affiliations:** Department of Parasitology, Faculty of Medical Sciences, Tarbiat Modares University, Tehran, Iran

**Keywords:** *Echinococcus granulosus*, EG95-1-6, epitope, GST, immunogenicity, P29, vaccine

## Abstract

Cystic echinococcosis (CE) is a neglected parasitic infection with a particular impact in humans and livestock. The current investigation was undertaken to design and evaluate a DNA vaccine encoding *Echinococcus granulosus* Eg95-1 to EG95-6, P29, and GST against hydatid cyst infection in BALB/c mice. Initially, B-cell, cytotoxic T-lymphocyte, and helper T-lymphocyte epitopes were forecasted using B-cell epitope prediction server (BCPREDS) and Immune Epitope Database (IEDB) server, respectively, and a vaccine construct incorporating multiple epitopes was rationally designed and comprehensively analyzed through in silico modeling and simulation studies. Next, *Escherichia coli* TOP10 was transformed by the recombinant pcDNA 3.1 plasmid and mass production, followed by plasmid extraction, was done. The BALB/c mouse immunization was done with 50 and 100 *μ*g concentrations of plasmid combined with IL-12 adjuvant or alone. Mouse sera and splenic lymphocytes were used for the measurement of specific humoral and cellular responses. The candidate vaccine model weighed 37.49 kDa with 338 residues antigenic, while nonallergenic, soluble, stable, highly thermotolerant, and hydrophilic in nature. Expression in HEK-293 cells was successfully achieved, as evidenced by the detection of a 37 kDa protein band in the western blot analysis. Vaccine doses, especially the 100 *μ*g concentration, alone or in combination with an adjuvant, induced a T-helper 1 (Th1)–type immune response. This was evidenced by higher levels of IgG2a antibody and interferon gamma (IFN-*γ*) along with lower levels of interleukin 4 (IL-4). Although the groups that received the 50-*μ*g dose of vaccine alone or with adjuvant showed a lower immune response, overall, the vaccinated groups showed statistically significant differences compared to the control groups (phosphate-buffered saline (PBS) and pcDNA). The promising results of this vaccine candidate can be further examined using challenges with various parasite genotypes.

## 1. Introduction

*Echinococcus granulosus* sensu lato (*E. granulosus* s.l.) is a zoonotic cestode species with a widespread distribution, present in canid intestinal tract, and it is the causative agent of cystic hydatid disease (CHD) or cystic echinococcosis (CE), a condition affecting humans and a broad range of other mammalian species [1]. According to the latest World Health Organization (WHO) roadmap, CE has been included within the 20 neglected tropical diseases (NTDs) worldwide [2]. In the areas of endemicity, such as central Asia, the Mediterranean Basin, and South America, the incidence of CE ranges between 1 and 200 per 100,000 individuals, particularly in poor rural communities having pastoral activities [3, 4]. Although the mortality remains to be low (2%–4%) [5], CE poses a remarkable burden to the health system, because of high treatment costs regarding chemotherapy and operational surgery [6]. Also, the disease negatively impacts on the productive and reproductive performances of affected farm animals, including reduced milk, meat, and wool production [7]. Environmental contamination of food/water resources by infected canids is the primary route of infection in intermediate (ungulates and lagomorphs) and/or incidental (humans) hosts [8]. The released oncosphere reaches the liver and lungs, which represent the most frequently affected organs, via circulation, and the development of hydatid cysts occurs very slowly throughout the years [9]. The parasite, also, demonstrates extensive genetic variation, and several genotypes have been confirmed for *E. granulosus* s.l. species cluster, including G1 and G3 as *E. granulosus* sensu stricto (*E. granulosus* s.c.), *Echinococcus equinus* (previously known as G4), *Echinococcus ortleppi* (formerly known as G5), G6/7 genotypic cluster, G8, and G10 as *E. canadensis*, as well as *Echinococcus felidis*. Of note, G2 and G9 are recognized as microvariants of G3 and G7, respectively [10]. Overall, diagnosis, treatment, and vaccine development against CE may be relatively affected by differences in antigenicity, infectivity, and cyst fertility of the above genotypes [11].

The CE remains a significant zoonotic infection in many parts of the world, in spite of regular dosing of dogs, enhanced slaughter hygiene, and the wide use of anthelmintics [12]. The increasing concern on drug-resistant parasites, due to long-term underdosing, and the occurrence of residual drug compounds in the meat and milk products derived from food animals subjected to therapeutic interventions emphasize the need for better, cost-effective control strategies, including vaccination programs [13]. An ideal vaccine against *E. granulosus* s.l. infections prevents the oncospheral evolution to hydatid disease in affected livestock and humans, while it may also inhibit the maturation of tapeworms in canids [7]. Thus, designing multivalent vaccines using crucial target antigens (reviewed in [14]) is of utmost medical and veterinary importance. Also, using different antigens would yield a more enhanced protective effect in experimental studies rather than single-antigen formulated vaccine candidates [15]. The EG95 protein is a 170-kDa molecule encoded by a multigene family (EG95-1 to EG95-7) and is very conserved among *E. granulosus* s.l. isolates. The sheep vaccine, EG95, is the most studied and evaluated vaccine against CE [16, 17], which has shown promising results based on clinical trials in China and South America, with up to 96% protection [18, 19]. P29 is a protoscolex and hydatid cyst antigen that possesses a pivotal role in host–parasite interaction, and the recombinant protein was shown to induce about 94.5% immunoprotection in sheep [20, 21]. Glutathione S-transferases (GSTs) represent a major group of multifunctional proteins directed towards detoxification of endogenous and/or exogenous toxic compounds, and some studies have shown protective effects exerted by vaccination using this protein [22, 23].

The target proteins chosen for vaccine development are not without limitations. For instance, P29 protein has been identified in the cyst germinal layer, whereas it is absent in extracts of adult worms. Additionally, regarding EG95, most research has concentrated on the G1 genotype. However, genetic differences, such as amino acid substitutions in the G6/G7 genotypes, may reduce the immunogenicity and effectiveness of the recombinant antigen. These variations underscore the importance of further exploring the EG95 gene family across diverse *E. granulosus* strains to enhance the vaccine's applicability and efficacy [24, 25].

The life cycle complexity of *E. granulosus* s.l. and its genetic diversity demand planning novel vaccination strategies [26], which can be reached by the utilization of stringent immunoinformatics (computational immunology) analyses [27]. Immunoinformatics assists us in accurate B- and T-cell-specific epitope screening and selection followed by construction of a potent vaccine candidate, for virulent antigenic proteins of a given pathogen in a cost- and time-effective manner [28]. This investigation was carried out with the purpose of developing a potential vaccine candidate through the design of a plasmid construct harboring the *E. granulosus* antigenic proteins Eg95-1 to Eg95-6, P29, and GST and subsequently assessing its protective capabilities against hydatid disease in the BALB/c mouse model.

## 2. Materials and Methods

### 2.1. In Silico Analyses

#### 2.1.1. Collecting Protein Sequences

For the immunoinformatics evaluation of the selected protein sequences and designing a multiepitope vaccine, the amino acid sequences of *E. granulosus* EG95-1 (Accession Number UKD60510.1), EG-95-2 (Accession Number AAG40126.1), EG95-3 (Accession Number AAG40128.1), EG95-4 (Accession Number AAG40124.1), EG95-5 (Accession Number AAG40125.1), EG95-6 (Accession Number AAG40123.1), P29 (Accession Number AHA85390.1), and GST (Accession Number AAD16438.1) were collected in FASTA format by the National Center for Biotechnology Information (NCBI), available at https://www.ncbi.nlm.nih.gov [29].

#### 2.1.2. Prediction of Immunogenic B- and T-Cell Epitopes

In order to predict continuous B-cell epitopes in the selected proteins, the B-cell epitope prediction server (BCPREDS) web server (http://crdd.osdd.net/raghava/bcepred) [30] was used, which provides three methods of prediction, including BCPred, ABCPred, and AAP. In this study, BCPred approach was used for prediction with a succession kernel-based support vector machine (SVM) classifier and > 0.75 cutoff score. The Immune Epitope Database and Analysis Resource (IEDB) was employed for the prediction of binding affinity between the selected peptides in protein sequences with mouse Class I (http://tools.iedb.org/mhci) (cytotoxic T-lymphocytes (CTL) epitopes) and Class II (http://tools.iedb.org/mhcii) (helper T-lymphocyte (HTL) epitopes) major histocompatibility complex (MHC) [31, 32]. Those epitopes having lower percentile ranks were anticipated to possess the highest affinity. A consensus method was selected to predict 13-mer MHC-I (H2-Db, H2-Dd, H2-Kb, H2-Kd, H2-Kk, and H2-Ld) and 15-mer MHC-II epitopes (H2-IAb and H2-IAd).

#### 2.1.3. Multiepitope Vaccine Construction

Those high-ranked HTL epitopes were chosen from each protein sequence to assemble the multiepitope vaccine construct. The epitopes were connected together using “SAPGTP” cleavable linker that enhances the segregation and processing of epitopes in the microcellular milieu. Also, a Kozak-like sequence was added to the N-terminal of the candidate vaccine sequence. Finally, the multiepitope vaccine construct consisted of six epitopic regions and GST gene complete (Table 1).

#### 2.1.4. Prediction of Physicochemical Properties and Posttranslational Modifications (PTMs)

Some of the major physicochemical features of the final construct, including grand average of hydropathicity (GRAVY), instability index, aliphatic index, extinction coefficients, evaluated in vitro and in vivo half-life, total number of positively and negatively charged residues, hypothetical isoelectric point (pI), molecular weight (MW), and number of amino acids, were forecasted using ExPASy ProtParam web server, available at https://web.expasy.org/protparam [33]. Moreover, some of the PTMs such as N-glycosylation, acylation, and phosphorylation were predicted for the multiepitope vaccine, using NetNGlyc (http://www.cbs.dtu.dk/services/NetNGlyc/) [34], NetAcet (http://www.cbs.dtu.dk/services/NetAcet/) [35], and NetPhos (http://www.cbs.dtu.dk/services/NetPhos/) [36] servers, respectively.

#### 2.1.5. Antigenicity, Allergenicity, and Solubility Prediction

Two web tools, ANTIGENpro (http://scratch.proteomics.ics.uci.edu) and VaxiJen v2.0 (http://www.ddg-pharmfac.net/vaxijen/VaxiJen/VaxiJen.html), were utilized for the forecasting of antigenicity. The latter server employs auto-cross-covariance (ACC) to generate vectors of major amino acid features from protein sequences. Also, ANTIGENpro provides a pathogen-independent prediction using protein microarray analysis outputs [37, 38]. In the following, the allergenicity of the final construct was evaluated using AlgPred server with 85% accuracy (threshold: 0.4) and based on a hybrid approach (http://crdd.osdd.net/raghava/algpred) [39]. The protein solubility was, also, forecasted upon overexpression via SOLpro web server (http://scratch.proteomics.ics.uci.edu) [40].

#### 2.1.6. Secondary and Tertiary Structure Predictions and Validation

The secondary structural characteristics of the final vaccine candidate construct were computationally elucidated and modeled using the Garnier–Osguthorpe–Robson (GOR) online web server and its bioinformatic algorithms (http://npsa-prabi.ibcp.fr/cgi-bin/npsa_automat.pl?page=npsa_gor4.html) [41]. Furthermore, a computation-aided homology modelling server, SWISS-MODEL, was utilized which predicts three-dimensional (3D) models of our multiepitope vaccine based on various templates (https://swissmodel.expasy.org) [42]. Additionally, to validate the fidelity of the predicted 3D structural model, a Ramachandran plot analysis was performed utilizing the PROCHECK online bioinformatic tool and its associated algorithms (https://saves.mbi.ucla.edu/) [43].

#### 2.1.7. Codon Optimization and In Silico Cloning

Codon optimization was done to enhance the proper expression of codons, based on the chosen (prokaryotic/eukaryotic) host. For this purpose, Java Codon Adaptation Tool (JCat) online server (http://www.jcat.de) was used to adapt the codons of the final vaccine model to *Escherichia coli* [44]. The output generated by the server is provided in the form of a graphical representation depicting the guanine–cytosine content percentage (GC%) and the codon adaptation index (CAI value). A CAI value between 0.8 and 1 and a GC% between 30% and 70% are optimum [45]. Moreover, NEBcutter v2.0 (http://nc2.neb.com/NEBcutter2) was selected to explore the cutting sites of a wide range of restriction enzymes within the codon-adapted sequence. In this study, *BamHI* and *EcoRI* restriction sites were embedded at the N-terminal and C-terminal of the nucleotide sequence of the vaccine. The finally codon-adapted protein was further ligated into the pcDNA3.1 plasmid vector and sent for construction and cloning into the pcDNA3.1 vector.

### 2.2. Transformation of *E. coli* TOP10 Strain by Recombinant pcDNA3.1

The recombinant pcDNA3.1 plasmid vector was maintained and amplified in the *E. coli* TOP10 strain cultivated in Luria–Bertani (LB) broth supplemented with 100 *μ*g/mL of the ampicillin antibiotic, with incubation carried out overnight at 30°C to facilitate vector propagation and enrichment. A colony PCR was done in a 25 *μ*L reaction, containing 2 *μ*l Master Mix (Ampliqon, Denmark), 1 *μ*L of each forward and reverse primer (10 pmol), 21-*μ*L distilled water (DW), and a separate grown colony on LB agar supplemented with 100 *μ*g/mL ampicillin. Also, the insertion of recombinant pcDNA3.1 in *E. coli* TOP10 bacteria was confirmed by sequencing of the purified plasmids and the results were blasted (https://blast.ncbi.nlm.nih.gov/Blast.cgi) with the reference sequences of each selected *E. granulosus* protein. Next, for large-scale plasmid extraction and purification, the Vivantis plasmid extraction kit (Vivantis Technologies, Malaysia) was used, based on the manufacturer's protocol.

### 2.3. Transfection and Storage of Human Embryonic Kidney (HEK)-293 Cells

For this purpose, purified plasmid DNA (25 *μ*g/mL) diluted in Tris-HCl (1:10) was combined with 100 *μ*L of CaCl_2_ solution (2.5 M) and adjusted to a final volume of 1 mL using sterile DW. Equal volumes of this solution were immediately mixed with 2X 4-(2-hydroxyethyl)-1-piperazineethanesulfonic acid (HEPES) buffer (274 mM NaCl, 10 mM KCl, 2.5 mM sodium phosphate dibasic heptahydrate (Na_2_HPO_4_-7H_2_O), 12 mM dextrose, and 42 mM HEPES) for a few seconds until turbidity indicative of agglutination was observed. The mixture was centrifuged at 16,000 × *g* for 30 s at 0°C. The pellet (100 *μ*L/1 mL culture medium) was added to HEK-293 cells that had been cultured overnight in Roswell Park Memorial Institute (RPMI) medium supplemented with fetal calf serum (FCS) enriched with 100 U/mL penicillin and 100 *μ*g/mL streptomycin at 37°C under 5% CO_2_. The incubation was carried out for 2–6 h. Subsequently, the cells were exposed to 20% glycerol in phosphate-buffered saline (PBS) for 1 min. After discarding the glycerol, freshly prepared medium was added, and incubation continued at 37°C under 5% CO_2_ for 1–3 days. Finally, the HEK-293 cells were washed with PBS, transferred to 1.5 mL microtubes, and stored at −20°C for further use.

### 2.4. Confirmation and Quantification of Vaccine Protein Production

The disruption of transfected HEK-293 cell membranes was achieved through consecutive freeze–thaw cycles with 4 *μ*L protease inhibitor, followed by five 1-min sonication steps. After centrifugation (3000 rpm, 5 min), the pellet was prepared for sodium dodecyl sulfate–polyacrylamide gel electrophoresis (SDS-PAGE) and western blotting. The pellet was mixed (3:1) with 4X sample buffer containing 2% *w*/*v* SDS, 0.05% *w*/*v* bromophenol blue, %*v*/*v*2-mercaptoethanol, 10% *v*/*v* glycerol, and 4.5 mM Tris-HCl; heated at 100°C for 5 min; and run on a 12.5% SDS-PAGE gel (80–120 V, 2 h) before staining with Coomassie Brilliant Blue R-250. For western blotting, proteins were transferred from the SDS-PAGE gel onto a nitrocellulose membrane (MilliporeSigma, United States) in transfer buffer (25 mM Tris (pH 8.3), 192 mM glycine, 20% methanol) overnight at 15–30 V. The membrane was blocked with bovine serum albumin (BSA) in tris-buffered saline with Tween-20 (TBS-T) (0.02 M Tris pH = 8.5, 0.5 M NaCl, 0.05% Tween-20) overnight at 4°C, then cut into 3-mm ribbons and incubated with 1–2 mL of patient sera at room temperature for 2–3 h. After three washes with TBS-T (10 min each), the ribbons were incubated with antihuman peroxidase conjugate (diluted 1:1000) for 2 h, followed by additional washes with TBS-T and PBS. Finally, diaminobenzidine (DAB) peroxidase (0.015% H_2_O_2_, 0.05% DAB, and 0.1 M PBS pH = 7.2) solution was applied to visualize the protein bands in brown [46].

### 2.5. Mass Extraction of the Recombinant pcDNA3.1 Plasmid Vector

At first, the recombinant bacteria were transferred to 50 mL LB medium with 50 *μ*g/mL ampicillin and incubated overnight in a shaking incubator at 37°C. Again, 1–2 mL of the cultivated bacteria were subcultured into a new LB medium and incubated at previous conditions. The cultures were centrifuged (13,000 rpm, 10 min, 4°C), the supernatant was discarded, and the pellet was mixed with 5 mL of Solution I (sterile mixture of 9.9 glucose, 3.94 g Tris-HCl, and 3.722 g EDTA in 1 L DW). In the following, 10 mL of Solution II (2 mL of 1 N NaOH, 1 mL of 10% sodium dodecyl sulfate (SDS), 7 mL sterile DW) was added and the tube was shaken well. After 8-min incubation on ice, 7.5 mL of Solution III (60 mL of 5 M acetate potassium, 11.5 mL glacial acetic acid, and 28.5 mL DW; filtered) and 30–40 *μ*L RNase A (stock: 10 mg/mL) were added and shaken well. Upon 20-min incubation on ice, centrifugation (13,000 rpm, 10 min, 4°C) was done and the supernatant was transferred to a 50-mL tube; then, equal volume of cold isopropanol was added, gently shaken, and incubated in a refrigerator for 10 min. The pellet was resuspended in 1-mL sterile DW, 500 *μ*L phenol, and 500 *μ*L chloroform were added and upon centrifugation (13,000 rpm, 10 min, 4°C), the supernatant (aqueous phase containing plasmid) was transferred to new sterile microtubes. This step was performed twice for higher yield of extracted plasmid. In the following, 3 M acetate sodium was added as 0.1 volume of the supernatants and cold absolute ethanol was added as two volumes of the supernatants. The microtubes were incubated overnight at −20°C, centrifugation was performed (12,000 rpm, 15 min), the supernatant was discarded, 200–300 *μ*L of cold 70% ethanol was added to each microtube then eluted, and the tubes were completely dried. Finally, 100-*μ*L sterile DW was added to resuspend the purified pelleted recombinant plasmids and kept at −20°C for further use.

### 2.6. In Vivo Experiment

#### 2.6.1. Animals

Female inbred BALB/c mice aged 6–8 weeks with a body weight of 20 ± 1 g were purchased from the animal breeding center of Razi Vaccine and Serum Research Institute. Standard conditions in terms of light, food, and water were provided for mice. The current study was approved by the Ethics of Tarbiat Modares University, Tehran, Iran (IR.MODARES.REC.1400.302).

#### 2.6.2. Immunization and Challenge

Seven experimental groups (10 mice/group) were sorted for current investigation, including (1) receiving PBS, (2) receiving empty pcDNA3.1 plasmid vector, (3) receiving only IL-12 genetic adjuvant, (4) 100 *μ*g recombinant plasmid without IL-12 genetic adjuvant, (5) 50 *μ*g recombinant plasmid without IL-12 genetic adjuvant, (6) 100 *μ*g recombinant plasmid with IL-12 genetic adjuvant, and (7) 50 *μ*g recombinant plasmid with IL-12 genetic adjuvant. The immunization experiment (100 *μ*L) was done three times with 2-week intervals (Days 0, 14, and 28) as intramuscular injection into the quadriceps muscle of mice using insulin syringe.

About 2–4 weeks after the last immunization, a number of 2000 viable *E. granulosus* protoscoleces (washed with PBS) were intraperitoneally injected to the vaccinated mice. These protoscoleces were extracted from sheep hydatid cysts isolated from local abattoirs, and their viability was validated through trypan blue staining. The challenged mice were maintained for about 8.5 months for hydatid cyst development, then euthanized using chloroform, and finally, the cyst sizes were measured using calipers, and the number of cysts was counted. Subsequently, comparisons were made between the different groups.

#### 2.6.3. Antibody Titers and Isotype Determination

In order to detect specific antibodies, the blood samples were collected randomly from five mice per each group at Days 14 and 28 postvaccination, the sera were separated and stored at −20°C until examined. The presence of specific IgG total, IgG1, and IgG2a antibodies in sera of BALB/c mice was assessed in triplicate, using commercial enzyme-linked immunosorbent assay (ELISA) kits from Karmania Pars Gene (Kerman, Iran) based on the manufacturer's instructions. The optical density (OD) of all samples was determined at 450 nm using ELISA reader.

#### 2.6.4. Cytokine Assay

To this end, 14 days after the last vaccination, three mice in each experimental group were euthanized and lymphocytes were harvested under aseptic conditions for cytokine evaluation [47]. The harvested lymphocytes were cultivated in RPMI-1640 medium (Gibco) supplemented with 20% fetal bovine serum (FBS). Next, 500 *μ*L of extracted lymphocyte suspension of each mouse was cultured in two wells of a 24-well cell culture plate, challenged with 50 *μ*g of crude antigen prepared [48], and the volume of each well was reached to 1 mL using complete RPMI medium. The plates were incubated for 48–72 h at 37°C with 5% CO_2_. Finally, the supernatant of each well was harvested and centrifuged (3000 rpm, 10 min); then, interferon *γ* (IFN-*γ*) and interleukin 4 (IL-4) cytokines were measured in triplicate by commercial ELISA kits (Karmania Pars Gene, Kerman, Iran), based on the manufacturer's protocol.

### 2.7. Statistical Analysis

The results of IgG total, IgG1, IgG2a, IFN-*γ*, and IL-4 were analyzed as mean and standard deviation (SD) for each experimental group using one-way ANOVA and Mann–Whitney statistics in SPSS statistics v.22. Also, the diagrams were drawn by Microsoft Excel and GraphPad Prism applications. The protection percentage was calculated using the following formula: 1 − (average number of cysts in immunized groups/average number of cysts in the control group) × 100.

## 3. Results

### 3.1. Computer-Based Vaccine Design and Validation

#### 3.1.1. Basic Characteristics of the Vaccine Model

**Figure 1:**
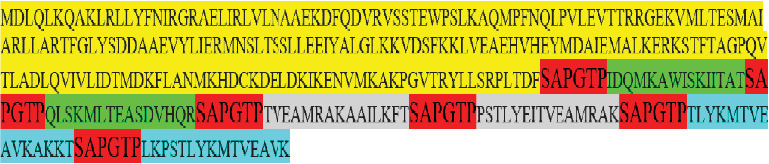
The final epitopes for DNA vaccine designed by several servers and use of linkers between them. GST2, complete gene (yellow); P29 (green); EG95/5 (gray); EG95/1, 2, 3, and 4 (blue); linker (red).

**Figure 2:**
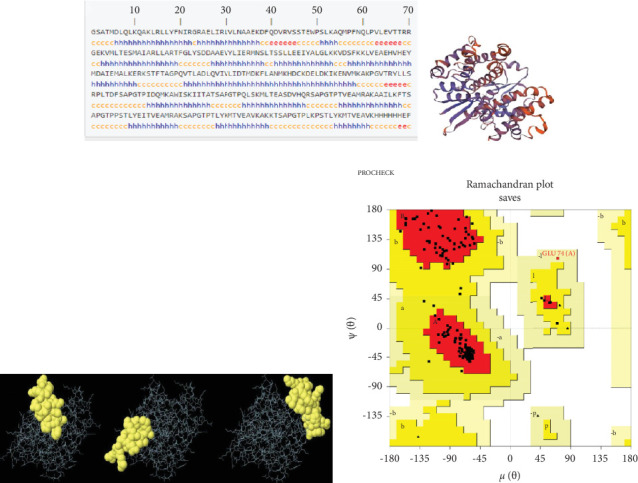
Structural analysis of the designed multiepitope vaccine candidate against cystic echinococcosis in mice. (a) Predicted secondary structure by the GOR-IV server (h = helix; e = extended strand; c = coil). (b) The predicted 3D model of the designed vaccine candidate protein, provided by the SWISS-MODEL server. (c) Conformational B-cell epitopes predicted using ElliPro tool of the IEDB server. (d) Ramachandran plot analysis of the 3D refined model, showing 93.4%, 6.1%, 0.5%, and 0.0% of the residues in the most favored, additional allowed, generously allowed, and disallowed regions, respectively.

Based on the epitope prediction results, those potent HTL (*n* = 6) epitopes were chosen for multiepitope vaccine construction, being connected together with “SAPGTP” linker (Figure 1). The final vaccine candidate model possessed a length of 338 amino acids and comprised *BamHI* and *EcoRI* cutting sites, the start/stop codon, and a Kozak-like sequence. This multiepitope vaccine candidate possessed a MW of 37.49 kDa, a relatively neutral pI (9.27), and over 30-h half-life in mammalian reticulocytes. Moreover, it was demonstrated to be a stable protein (instability index: 32.36), having high thermotolerance (aliphatic index: 90.68), and it was hydrophilic in nature (GRAVY: −0.177). The designed vaccine had single acetylation (position: 3), 0 N-glycosylation, and 53 phosphorylation sites (Table 2). Alpha helix (52.07%) was the most prominent secondary structure in the vaccine model, followed by random coil (30.77%), extended strand (10.65%), and beta turn (6.51%) (Figure 2(a)). The vaccine candidate model was predicted using the SWISS-MODEL web server (Figure 2(b)), and three conformational B-cell epitopes were predicted with the following properties: (i) 17 residues, score: 0.813; (ii) 11 residues, score: 0.784; and (iii) 21 residues, score: 0.763 (Figure 2(c)) was further refined, and the Ramachandran plot analysis showed that 93.4%, 6.1%, 0.5%, and 0.0% of the residues were assigned to the most favored, additional allowed, generously allowed, and disallowed regions, respectively (Figure 2(d)). Additionally, the vaccine candidate model was found to be antigenic (antigenicity score: 0.4048), nonallergenic, and relatively soluble (solubility score: 0.527) in nature, as predicted by VaxiJen v2.0, AlgPred, and SOLpro web servers, respectively. Codon optimization results showed that the CAI value and GC% were improved from 0.62% and 45.85% before adaptation, respectively, to the 1% and 50.61% after codon adaptation. Finally, the multiepitope fragment with the above desired properties was synthesized by a gene synthesis company. The resulting synthetic construct was subsequently used as an immunogen in subsequent steps.

### 3.2. In Vivo Experiments

#### 3.2.1. Protein Expression

**Figure 3:**
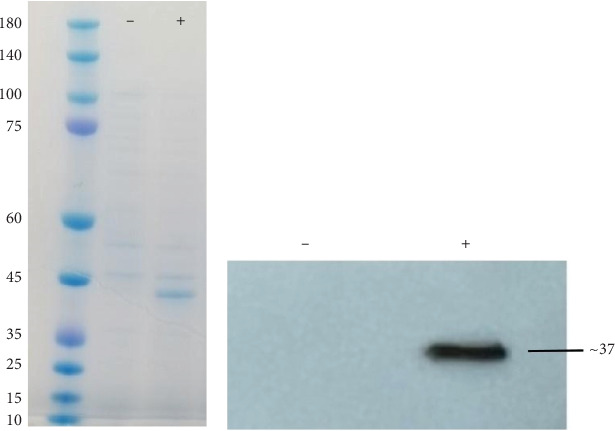
Protein expression. (a) SDS-PAGE shows a prominent band in the 35–40 kDa range. (b) Western blot analysis confirms the presence of a protein with a molecular weight of 37 kDa. Positive: transfected cells containing multiepitope vaccine; negative: transfected cells containing pcDNA3.1 (negative control).

The results of the SDS-PAGE analysis revealed that the protein expressed from the plasmid in transfected HEK-293 eukaryotic cells had a molecular weight within the range of 35–45 kDa (Figure 3(a)). This was further confirmed through western blot analysis, which identified a distinct band of approximately 37 kDa using an antihistidine tag antibody (Figure 3(b)).

#### 3.2.2. Measurement of Humoral Immune Responses

**Figure 4:**
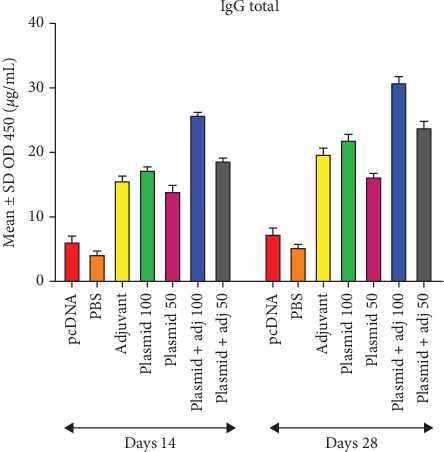
Mean ± SD of the obtained ODs (450 nm) regarding IgG total levels in the experimental groups in Days 14 and 28 (micrograms per milliliter).

**Figure 5:**
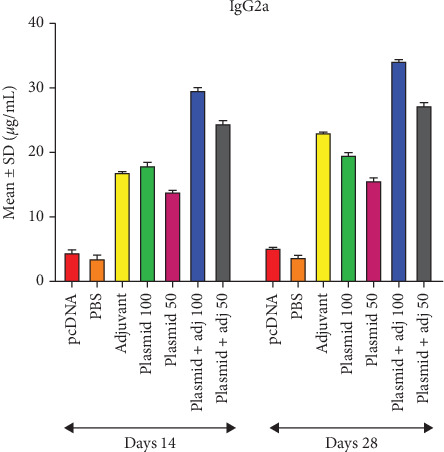
Mean ± SD of the obtained ODs (450 nm) regarding IgG2a levels in the experimental groups in Days 14 and 28 (micrograms per milliliter).

**Figure 6:**
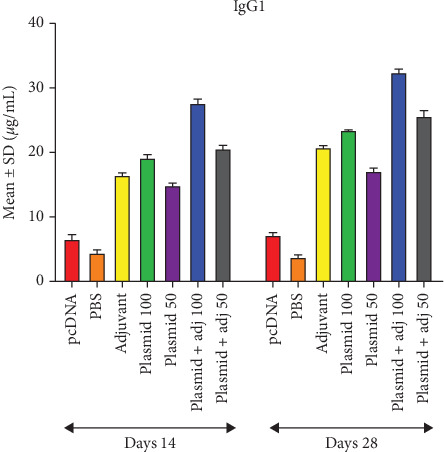
Mean ± SD of the obtained ODs (450 nm) regarding IgG1 levels in the experimental groups in Days 14 and 28 (micrograms per milliliter).

With respect to the measurement of total IgG, the highest OD during two bleeding timepoints belonged to those mice receiving 100 *μ*g plasmid + adjuvant, followed by those receiving 50 *μ*g plasmid + adjuvant and 100 *μ*g plasmid alone as well as adjuvant alone. Of note, the lowest OD was associated with the first (Day 14) and second (Day 28) bleeding timepoints of the PBS group (Figure 4). Similar results were found regarding IgG2a titer estimation, except for the last two lowest ODs, belonging to the adjuvant along and 100 *μ*g plasmid alone, respectively (Figure 5). Also, the trend of IgG1 was similar to that of total IgG (Figure 6).

#### 3.2.3. Measurement of Cell-Mediated Responses

**Figure 7:**
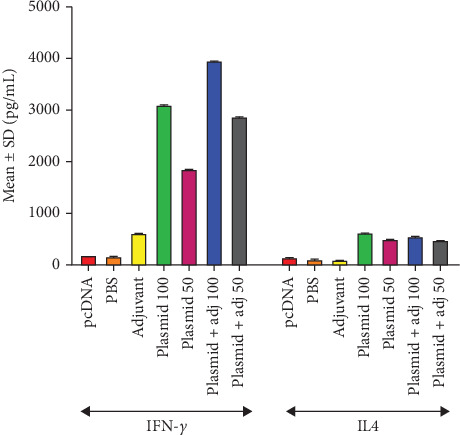
Mean ± SD of IFN-*γ* and IL-4 levels in the experimental groups (picograms per milliliter).

Based on our results, the PBS and then pcDNA3.1 groups had the lowest rates, while the highest IFN-*γ* belonged to the 100 *μ*g plasmid + adjuvant, followed by 50 *μ*g plasmid + adjuvant and 100 *μ*g plasmid alone. Moreover, the highest IL-4 levels were estimated for those mice receiving 100 *μ*g plasmid alone, while it was the lowest in adjuvant alone group (Figure 7).

#### 3.2.4. Protoscolex Challenge and Cyst Measurements

In this study, about 8.5 months after the intraperitoneal challenge of BALB/c mice with protoscoleces, the animals were killed and dissected, and the formed cysts were counted and measured with a caliper. The mean cyst dimensions for each group are presented in Table 3. In most of the vaccinated groups, only a small number of hydatid cysts of small sizes were formed, whereas mice in the control group developed numerous cysts of significantly larger sizes (supporting figure (available here)). The highest percentage of protection was estimated for mice that received 100 *μ*g of plasmid + adjuvant, compared to mice that received 50 *μ*g of plasmid + adjuvant and 50 *μ*g of plasmid alone (Table 3).

## 4. Discussion

CE is a neglected parasitic infection, mostly in countries heavily involved in raising livestock, which inflicts considerable disease burden and disability-adjusted life years (DALYs) to the affected people as well as substantial costs to the livestock husbandry [2, 49]. Thus, immunoprophylaxis seems to be a rational preventive strategy for this infection. During last decades, various single- and multiple-component vaccine candidates have been devised and examined against CE infection [14]. The EG-95 gene family encodes a 17 kDa protein containing a secretory signal peptide, a hydrophobic motif, and a fibronectin Type III domain [50]. It is believed that the latter domain permits the oncosphere to connect with different receptor types within host, as likely evidence in the ability of oncosphere to infect different hosts [51]. The P29 protein is a common protoscolex antigen, being absent in the adult worms [52]; it has been shown to possess in the host–parasite interactions and seem to be a good candidate for diagnostic and/or vaccination purposes [21, 53]. Another protoscolex protein, GST, is known as a potent immunomodulatory, favoring parasite persistence within the host tissues [54]. By the advent of recent millennium, a novel branch of bioinformatics emerged, called immunoinformatics, directed towards utilization of web-based and/or standalone tools for the prediction of B- and T-cell-specific immunodominant components of a given antigenic protein, that is, epitopes, and subsequent engineering and construction of multiepitope vaccine candidates against the respective pathogens. This approach may actually overcome some of the shortcomings of the traditional vaccine design, regarding experimental costs and being time-consuming [28]. The current investigation was done to design a potential vaccine candidate using a plasmid encoding *E. granulosus* Eg95-1 to EG95-6, P29, and GST and evaluated its protective efficacy against hydatid disease in BALB/c mice.

At the first step, we performed epitope analysis using the IEDB web server for each selected *E. granulosus* s.l. protein (EG-95, GST, and P29); based on the lower percentile ranks (higher affinity for mouse MHC binding), six HTL epitopes and GST complete gene were selected. The final vaccine candidate designed using these epitopes possessed 338 residues. A good vaccine candidate should be highly antigenic, while nonallergenic in nature [55], which was the case for our designed model. Also, based on the ProtParam server output, the designed vaccine had a 37.49 MW, being a likely immunogen (proteins over 5–10 kDa are potential immunogens), and it was a stable molecule (instability index: 32.36) with high thermotolerant ability (aliphatic index: 90.68) and high interactions with the surrounding water milieu (GRAVY: −0.177). Finally, this multiepitope protein was sent for the production of a DNA-based vaccine (in pcDNA 3.1 vector), and its in vivo efficacy as formulated with or without IL-12, as a genetic adjuvant, was demonstrated in BALB/c mice.

Immunity against CE involves both pre- and postformation of hydatid cysts. Initially, levels of IgG antibodies are increased 2 weeks (in mice) or 11 weeks (in sheep) upon protoscolex or parasite egg challenge [56, 57]. Such antioncospheral antibodies play a significant role in parasite killing and protection of the host against echinococcosis [58]. In chronic infection courses, the levels of IgG, IgM, and IgG are increased. Cell-mediated immunity, also, is stimulated in the initial infection phase, which increases as the infection progresses [59–61]. Notably, both T-helper 1 (Th1)-type (IFN-*γ* and IL-12) and Th2-type (IL-4, IL-5, and IL-10) responses are observed in CE infection, probably being associated with the type and the volume of the released antigens [62]. It is known that IgE and IgG4 responses are elicited by IL-4, while IFN-*γ* and eosinophilia are stimulated through IL-3 and IL-5 [63, 64]. Progressive growth of a hydatid cyst or protection of the host against it is directly associated with the predominance of a given Th response [65]. The production of IgG2 is related to the IFN-*γ*-mediated B-cell activation, while other antibody classes are impacted by IL-4 and IL-5 cytokines [66]. Altogether, Th1-type responses favor host protection against hydatid cyst growth and this should be considered in vaccine design studies against CE. The results of BALB/c mouse immunization using our designed multiepitope vaccine showed that the specific IgG levels significantly increased in the vaccinated mice, in comparison with control groups (*p* < 0.05), being consistent with previous studies using vaccine candidates against echinococcosis [20, 47, 67, 68]. The plasmid vaccine elicited both Th1/Th2 responses, with the predominance of IgG2a (Th1-type), rather than IgG1 (Th2-type) responses, causing hydatid cyst elimination in the vaccinated groups. Also, the elevated rate of IgG2a and IgG1 in all vaccinated groups was shown to have statistically significant difference with control groups (*p* < 0.05).

In this study, also, the bias in cellular immunity towards Th1 or Th2 was measured in splenic lymphocytes using ELISA method. The average levels of IFN-*γ* in the vaccinated groups were reported to be 3108 (100 *μ*g plasmid), 1860 (50 *μ*g plasmid), 3959 (100 *μ*g plasmid + adjuvant), and 2882 (50 *μ*g plasmid + adjuvant) pg/mL, showing statistically significant difference with control groups (*p* < 0.05). It is, also, noteworthy that the mean IL-4 levels in 100 *μ*g plasmid (631.1 pg/mL), 50 *μ*g plasmid (502 pg/mL), 100 *μ*g plasmid + adjuvant (564.7 pg/mL), and 50 *μ*g plasmid + adjuvant (489 pg/mL) groups differed significantly with those in control groups. Of note, the levels of IFN-*γ* and IL-4 in adjuvant alone group (622.7 vs. 97.33 pg/mL) was relatively higher and lower, respectively, than those in controls, which may result from the nature of IL-12 genetic adjuvant, causing the IFN-*γ* upsurge, while reducing IL-4 by itself. It is said that genetic adjuvants can efficiently promote the immune induction by DNA vaccines, improve the antigen uptake by antigen-presenting cells (APCs), and prevent from enzymatic degradation of the vaccine candidate [13, 69, 70].

## 5. Conclusion

The dog tapeworm, *E. granulosus* s.l., poses a significant threat to the human and livestock populations by inducing hydatid cysts. Therefore, vaccination approaches are necessary to prevent this parasitic infection. The study at hand entailed the rational design of a multiepitope vaccine using EG-95_1-6_, P29, and GST proteins of *E. granulosus* s.l., and its efficacy was examined in two experimental doses (50 and 100 *μ*g) combined with IL-12 genetic adjuvant and alone in BALB/c mice against CE infection. The mean levels of IFN-*γ* and IgG2a, as representatives of Th1-type immunity, were substantially higher in vaccinated than control groups. This was evidenced by no hydatid cyst formation or the growth of few, small-sized cysts in the vaccinated BALB/c mice and highlights the adequate immunogenic potential of this vaccine candidate. It is recommended to examine the vaccine candidate efficacy against different parasite genotypes, and employ different doses formulated with other potent genetic adjuvants.

## Figures and Tables

**Table 1:** Utilized *E. granulosus* s.l. genes, proteins, and peptides for vaccine design in the present study.

**Gene**	**Method**	**Start**	**End**	**Protein/peptide**	**Length**	**Percentile rank**	**Reference**
GST2	Complete CDS protein	—	—	>AAD16438.1 glutathione S-transferase [*Echinococcus granulosus*] MAPTLAYWDIRGLAEQSRLLLKYLEVEYDDKRYKIGSTPTFDRSAWLSEKFSLGLDFPNLPYYIDGDFKLTQSGAILEYIADRHGMIPDCKKRRAVLHMLQCEVVDLRMAFTRTCYSPDFEKLKPGLFETLAQKLPNFEAYLGEKEWLTGDKINYPDFSLCELLNQLMKFEPTCLEKYPRLKAYLSRFENLPALRDYMASKEFKTRPCNGASAKWRGDC	219	—	[71]
P29	IEDB MHCII	40	54	IDQMKAWISKIITAT	15	0.0098	Current study
P29	IEDB MHCII	100	114	QLSKMLTEASDVHQR	15	0.0095	Current study
EG95-5	IEDB MHCII	103	117	TVEAMRAKAAILKFT	15	0.0227	Current study
EG95-5	IEDB MHCII	96	110	PSTLYEITVEAMRAK	15	0.0760	Current study
EG95-1,2,3,4	IEDB MHCII	98	112	TLYKMTVEAVKAKKT	15	0.0366	Current study
EG95-1,2,3,4	IEDB MHCII	94	108	LKPSTLYKMTVEAVK	15	0.0098	Current study

**Table 2:** Prediction of physicochemical parameter and PTM sites of the designed vaccine candidate.

**Parameter**	**Results**	**Tools**	**Reference**
No. of residues	338	ProtParam	[72]
MW	37.49 kDa		
Speculated pI	9.27		
Estimated half-life	>30 h		
Instability index	32.36		
Aliphatic index	90.68		
GRAVY	−0.177		
Signal peptide	No	SignalP-4.1	[72]
N-Glycosylation sites	0	NetNGlyc 1.0	[72]
Phosphorylation sites	53	NetPhos 3.1	[72]
N-Terminal acetylation	1	NetAcet 1.0	[72]

**Table 3:** The number and size of detected hydatid cysts during postmortem abdominal examination per study groups and the calculated protection percentage based on vaccination protocol.

**Variables**	**Experimental groups**						
	**PBS**	**PcDNA**	**Adjuvant**	**Plasmid (100 *μ*g)**	**Plasmid (50 *μ*g)**	**Plasmid (100 *μ*g) + adjuvant**	**Plasmid (50 *μ*g) + adjuvant**
Free cysts in peritoneal cavity	70	68	57	5	6	2	3
Cysts attached to organs	109	95	72	13	18	4	7
Mice with/without cyst	4/1	5/0	4/1	2/3	3/2	1/4	2/3
Average size of cyst (cm)	1.3	1.09	0.98	0.7	0.8	0.3	0.5
Protection percentage^a^	0%	0%	28%	90%	87%	96.7%	94.5%

^a^Protection percentage: 1 − (average number of cysts in immunized groups/average number of cysts in control group) × 100.

## Data Availability

The entire required data are available and recorded in the format of text we have attached as supporting information files (supporting data).

## Supporting Information

Supporting Information Additional supporting information can be found online in the Supporting Information section. Supporting figure: It shows experimental mice in different treatment groups. Figures A, B, and C show mice from control groups that received PBS or empty pcDNA vector. In contrast, Figures D, E, and F show mice belonging to the experimental vaccinated groups that received the multiepitope DNA vaccine construct.
