# Supplementary material for: Designing and Evaluation of a Plasmid Encoding Immunogenic Epitopes From Echinococcus granulosus Eg95-1-6, P29, and GST Against Hydatid Cyst in BALB/c Mice
Source: J Parasitol Res. 2025 Feb 24;2025:1655679. doi: 10.1155/japr/1655679 (PMC11876539; doi:10.1155/japr/1655679)
Supplement: Supporting Information — Additional supporting information can be found online in the Supporting Information section. Supporting figure: It shows experimental mice in different treatment groups. Figures A, B, and C show mice from control groups that received PBS or empty pcDNA vector. In contrast, Figures D, E, and F show mice belonging to the experimental vaccinated groups that received the multiepitope DNA vaccine construct. [file 1655679.f1.docx]

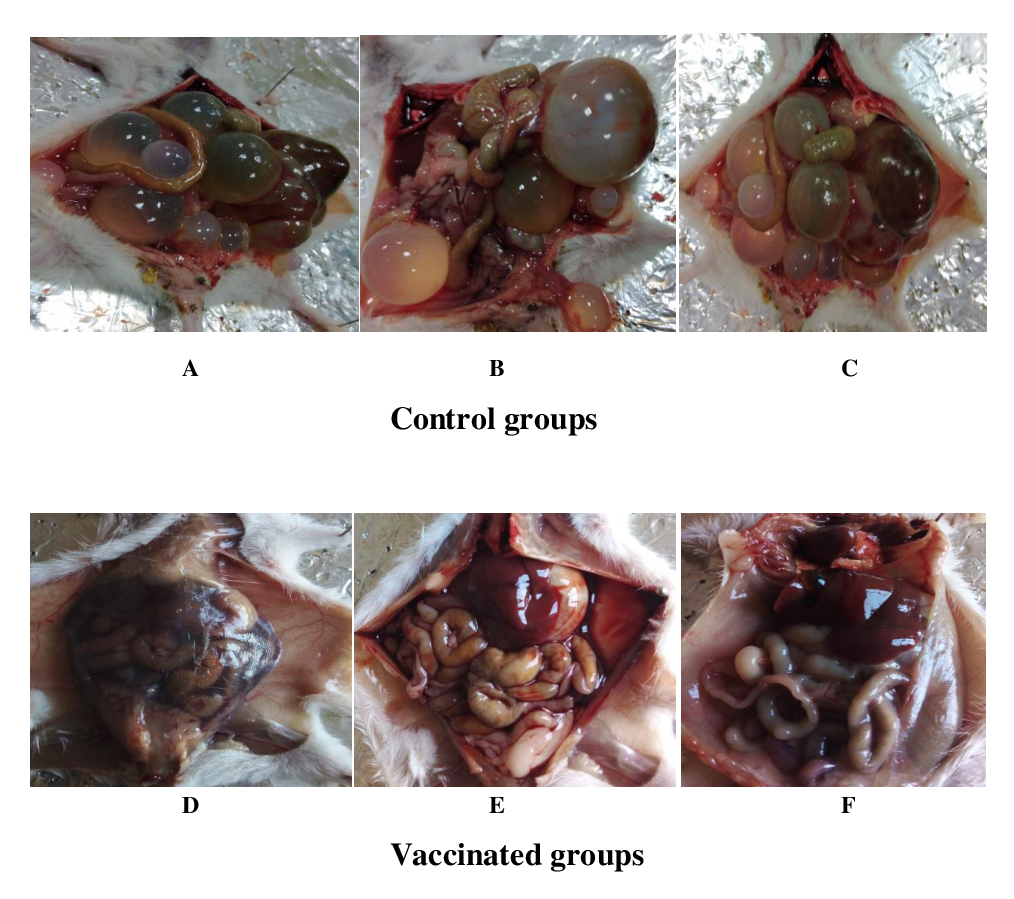


**Supplementary figure.** It shows experimental mice in different treatment groups. Figures A, B, and C show mice from control groups that received PBS or empty pcDNA vector. In contrast, Figures D, E, and F show mice belonging to the experimental vaccinated groups that received the multi-epitope DNA vaccine construct.
